# Chondroitin sulfate regulates proliferation of *Drosophila* intestinal stem cells

**DOI:** 10.1371/journal.pgen.1011686

**Published:** 2025-05-09

**Authors:** Collin Knudsen, Ayano Moriya, Eriko Nakato, Rishi Gulati, Takuya Akiyama, Hiroshi Nakato

**Affiliations:** 1 Department of Genetics, Cell Biology, and Development, University of Minnesota, Minneapolis, Minnesota, United States of America; 2 Department of Biology, The Porter Cancer Research Center, Indiana State University, Terre Haute, Indiana, United States of America; Indian Institute of Science Education and Research Mohali, INDIA

## Abstract

The basement membrane (BM) plays critical roles in stem cell maintenance and activity control. Here we show that chondroitin sulfate (CS), a major component of the *Drosophila* midgut BM, is required for proper control of intestinal stem cells (ISCs). Loss of *Chsy*, a critical CS biosynthetic gene, resulted in elevated levels of ISC proliferation during homeostasis, leading to midgut hyperplasia. Regeneration assays demonstrated that *Chsy* mutant ISCs failed to properly downregulate mitotic activity at the end of regeneration. We also found that CS is essential for the barrier integrity to prevent leakage of the midgut epithelium. CS is known to be polymerized by the action of the complex of Chsy and another critical protein, Chondroitin polymerizing factor (Chpf). We found that *Chpf* mutants show increased ISC division during midgut homeostasis and regeneration, similar to *Chsy* mutants. As *Chpf* is induced by a tissue damage during regeneration, our data suggest that Chpf functions with Chsy to facilitate CS remodeling and stimulate tissue repair. We propose that the completion of the repair of CS-containing BM acts as a prerequisite to properly terminate the regeneration process.

## Author summary

The control of stem cells is important to many areas of developmental and cell biology, including cancer, stem cell therapies, and tissue regeneration following injury. The *Drosophila* midgut, closely homologous to the human small intestine, is a model that allows for detailed investigation of the intestinal stem cells (ISCs). We used the midgut to study the impact of losing chondroitin sulfate (CS) in the digestive system. CS is an extracellular matrix component and is present in the basement membrane of *Drosophila* midguts. We found that a lack of CS results in higher levels of proliferation under homeostatic and regenerative conditions. Furthermore, CS functions in protecting the gut barrier, helping to keep the interior of the gut from the inside of the body. This study highlights the importance of CS and the overall basement membrane in controlling midgut morphology, ISC proliferation, and the midgut’s response to stressors such as infection or aging.

## Introduction

The basement membrane (BM) is a thin and dense sheet of specialized extracellular matrix (ECM) that underlies epithelial cell layers and surrounds most tissues in all metazoans [1]. Major BM components include type IV collagen, laminin, nidogen, and the heparan sulfate proteoglycan (HSPG) perlecan [2]. The BM acts as a physical restraint to form tissue architecture [3–5], or as an extracellular scaffold, tethering growth factor ligands, such as TGF-β/BMP, FGF, Hedgehog (Hh), and Wingless (Wg)/Wnt [6–10]. Thus, the BM regulates tissue patterning and organ shape by integrating mechanical and biochemical signaling [2].

The BM plays a critical role in regulating the *Drosophila* midgut intestinal stem cells (ISCs) [11,12]. The *Drosophila* midgut consists of a single layer epithelium with two types of differentiated cells, absorptive enterocytes (ECs) and secretory enteroendocrine cells (EE). ISC divisions produce precursor cells, the enteroblasts (EBs), and the enteroendocrine precursors (EEPs) that differentiate into ECs and EE, respectively [13–17]. The midgut epithelium is surrounded by a BM and visceral muscle. ISCs are located sporadically throughout the intestinal epithelium and are directly in contact with the BM [18,19]. The BM components affect ISC proliferation during midgut homeostasis. For example, loss of perlecan causes ISCs to detach from the BM, leading to the loss of stem cell identity [11]. Furthermore, type IV collagen concentrates Dpp to the basal surface, supporting the ISCs to maintain their stemness [12]. Several integrin subunits are enriched at the interface of ISC/EB and the BM [20], and these molecules regulate ISC asymmetric division, proliferation, and maintenance [20–23]. Thus, BM components and integrin-mediated cell-BM adhesion plays a critical role during midgut homeostasis.

The ISC model offers a powerful system to study molecular mechanisms of regeneration [24–26]. Midgut regeneration can be induced by feeding flies damage-inducers, such as the bacteria *Erwinia carotovora* strain 15 (*Ecc15*) [24,27] or *Pseudomonas entomophila* (*Pe*) [25,28] and the compound dextran sodium sulfate (DSS) [29–31]. In response to midgut damage, the epithelium activates several growth factor pathways to promote ISC proliferation [25,29]. These damage-induced mitogenic factors include Unpaired 3 [25,27,32,33], Hh [34], Wg [35], BMP/Dpp [36–38], and Vein, an EGFR ligand [39,40]. Once the regeneration is completed, these mitogenic pathways are downregulated and return to a homeostatic state. There are two key questions regarding this regeneration termination. First, how does a tissue recognize when the regeneration is complete? For example, what acts as a prerequisite for the termination process? Second, how does the tissue downregulate stem cell mitotic activity to return to the homeostatic state? Recent studies have identified some molecules that negatively regulate ISC division [41–44]. However, the prerequisite factors that permit shutdown of ISC proliferation at the end of regeneration remain to be determined.

Chondroitin sulfate (CS) is an evolutionary conserved glycosaminoglycan found in most animal species, including *Drosophila*. CS chains are attached to specific serine residues of core-proteins, forming proteoglycans (PGs). Some CSPGs are secreted and embedded into the ECM and others are membrane proteins, functioning on the cell surface. CS is present in high quantities in the BM and has important biological functions across animal species [45–47]. To investigate the functions of CS during development, we recently established a CS-deficient model in *Drosophila*: mutants for *Chondroitin sulfate synthase* (*Chsy*). *Chsy* encodes the *Drosophila* homologue of mammalian Chondroitin sulfate synthase-1 (Chsy-1), a critical CS biosynthetic enzyme [48]. Despite a complete lack of CS, a fraction of *Chsy* mutants survive to the adult stage. In the mutant ovary, the initial assembly of the organ can occur relatively normally in the absence of CS. However, *Chsy* mutants exhibited altered BM stiffness and abnormal muscle structure and function, leading to a gradual degradation of the gross organ structure as mutant animals aged.

In this study, we examined the role of CS in ISC control. Analyses of the *Drosophila* mutants *Chsy* and *Chpf* indicated that CS plays a key role in controlling ISC proliferation during both homeostasis and regeneration. CS is also required for midgut barrier integrity. Based on our observations, we propose a model that CS remodeling is stimulated by *Chpf* induced during regeneration and acts as a permissive cue to proceed to the downregulation of ISC division.

## Results

### Production of midgut basement membrane CS

**Fig 1:**
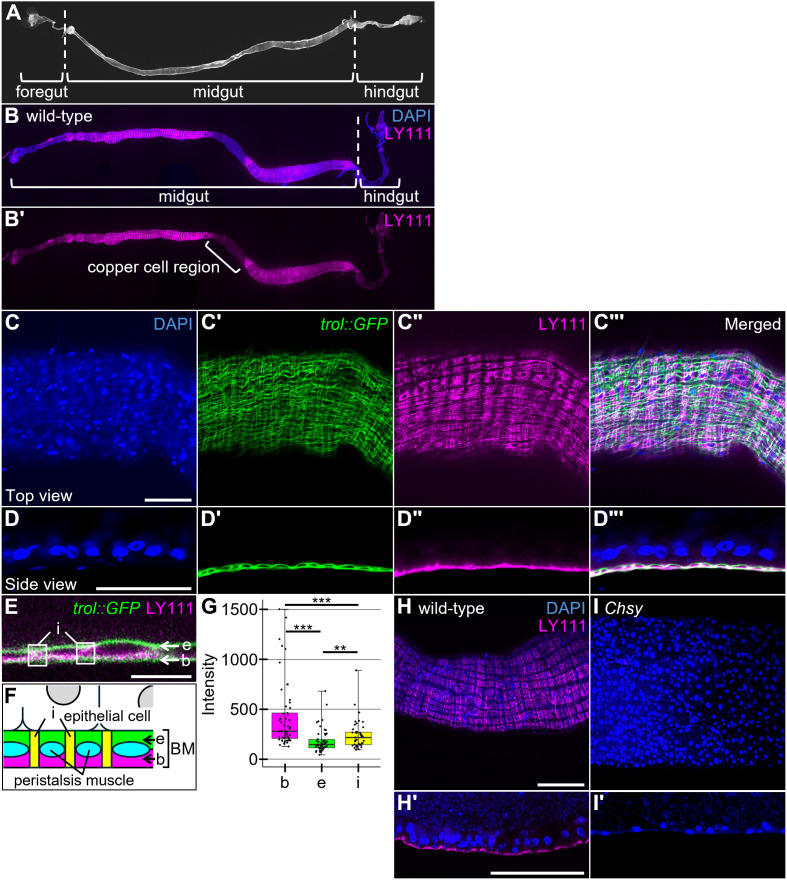
CS distribution in midgut. (A) Anatomical structure of the adult digestive tract. The digestive tract is composed of three main regions: foregut, midgut, and hindgut. (B-B’) Distribution of CS (LY111 antibody; magenta) throughout the entire foregut, midgut, and hindgut of wild-type flies. The copper cell region is marked by the bracket. (C-D’“) The lateral (C-C’”) and transverse (D-D’”) views of *trol::GFP* (green) midguts stained with LY111 (magenta) and DAPI (blue). (E-G) Uneven distribution of CS in the midgut. (E) A high magnification view focusing on the BM of the midgut stained for *trol::GFP* (green) and LY111 (magenta). CS distribution is present in the BM with a staining pattern distinct from the major BM component Perlecan (encoded by *trol*). We classified the LY111 signal into three different regions in the BM: 1) the basal side of the BM, which is outer layer of the peristalsis muscles (marked as b), 2) epithelial side (marked as e), and 3) inter-muscle region, which is the gap space between the peristalsis muscle (marked as i). (F) A diagram showing the regions b (magenta), e (green), and i (yellow). The peristalsis muscle is shown in light blue. (G) Quantification of CS localization in the BM. CS is more densely observed at the basal side of the BM. (H-I’) LY111 staining of *Chsy* mutant midguts. Wild-type (H and H’) and *Chsy* mutant (I and I’) midguts stained with LY111 (magenta) and DAPI (blue). CS is undetectable in *Chsy* mutants. Boxes indicate the 25–75th percentiles, and the median is marked with a line. The whiskers extend to the highest and lowest values within 1.5 times the interquartile range. **P < 0.01; ***P < 0.001 (two-sided, unpaired *t*-tes*t*). Scale bars: 100 μm (C, D, H, and H’); 10 μm (E).

In our recent study, anti-CS antibody (LY111) staining of the adult ovary revealed that CS is mainly localized in the BM [48]. Using the same antibody, we examined CS distribution in the adult digestive system (Fig 1). Anti-CS staining signal was detected broadly except the middle midgut region near the copper cells that lacks the LY111 signal (Fig 1A–1Bi). In the posterior midgut, CS is specifically observed in the BM, overlapping with the *trol::GFP* marker (Fig 1C–1E). *trol* encodes the *Drosophila* orthologue of perlecan, a principal constituent of the BM, [49,50] and *trol::GFP* is widely used as a BM marker [9,41,51–53]. Co-staining of LY111 with *trol::GFP* showed uneven distribution of CS within the BM: CS is more densely observed at the basal side of the BM (b), outer layer of the peristalsis muscles, compared to the epithelial side (e) or inter-muscle region (i), the gap space between the peristalsis muscle (Fig 1E–1G). We believe that there are transmembrane CSPGs expressed in the ECs, including Wdp [54]. However, no significant co-localization of LY111 staining and membrane-bound GFP (*MyoIA>membrane-GFP*) was detectable (S1 Fig), suggesting that midgut CS most abundantly exists in the BM. We found that CS is completely undetectable in *Chsy* mutants (Fig 1H–1Ii), confirming the essential role of *Chsy* in midgut CS biosynthesis.

**Fig 2:**
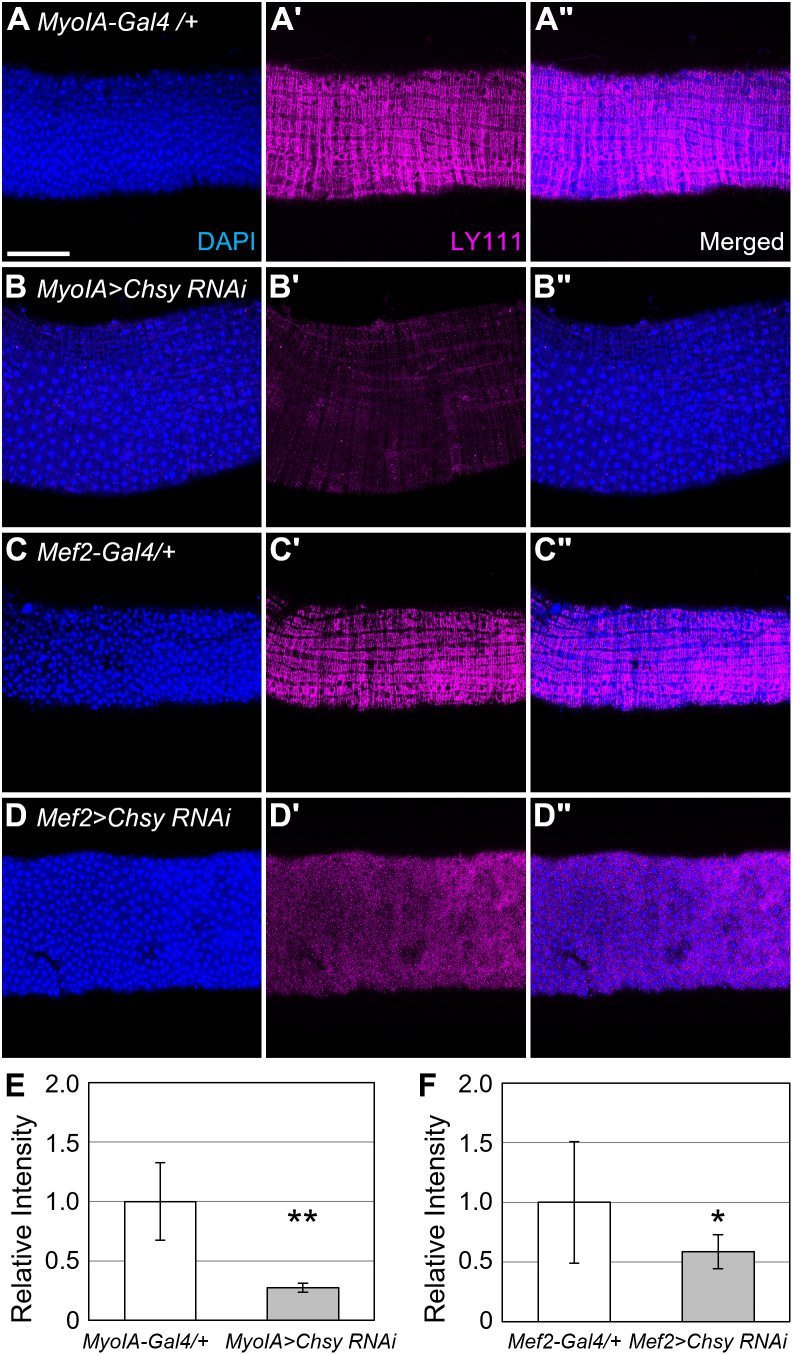
Midgut CS originates from enterocytes and muscle. (A-D“) Midguts were stained with DAPI (blue) and LY111 (magenta) to investigate the source of CS in the midguts. *MyoIA-Gal4* (A-B”) and *Mef2-Gal4* (C-D”) were crossed with *UAS-Chsy RNAi* to knockdown CS production in ECs (B-B”) and in muscle (D-D”), respectively. *MyoIA-Gal4/+* (A-A”) and *Mef2-Gal4/+* (C-C”) were used as controls. *MyoIA-Gal4* flies were under temperature control and flies were shifted to drive expression 2 days post egg laying. (E and F) Quantification of LY111 staining by *Chsy* knockdown in ECs (E) and muscle (F). The average fluorescence intensity of the control (*MyoIA-Gal4/ +* or *Mef2-Gal4/+*) was normalized to 1.0. *P < 0.05; **P < 0.01 (two-sided, unpaired *t*-tes*t*). Scale bar: 100 μm.

Previous studies have shown that some BM components are synthesized locally within the organ and others are produced in a different organ (e.g., fat body), which are transported via hemolymph [55,56]. To determine the source of the CS found in the midgut, we examined the effects of expression of a *UAS-Chsy RNAi* transgene with various cell type-specific Gal4 drivers, *esg-Gal4* (ISCs and EBs), *MyoIA-Gal4* (ECs), *Mef2-Gal4* (muscle), *pros-Gal4* (EEs), and *Lpp-Gal4* (fat body) (Figs 2 and S2). Anti-CS staining was significantly reduced when *Chsy RNAi* was induced by *MyoIA-Gal4* (Fig 2A–2Bii) and *Mef2-Gal4* (Fig 2C-2Dii). Quantitative analyses showed that LY111 staining was reduced to 27% and 59% of the wild-type level in *MyoIA>Chsy RNAi* and *Mef2 > Chsy RNAi* animals, respectively (Fig 2E and 2F). In contrast, *Chsy* knockdown using other Gal4 drivers did not affect the anti-CS signal (S2A–S2Cii Fig). These results demonstrated that midgut CS originates locally, mainly from the ECs and visceral muscle as the source.

To analyze the timing of the local production of CS by the ECs, we used the TARGET system, which provides temporal control of cell-type specific gene expression [57]. Using the genotype of *MyoIA-Gal4 tub-Gal80*^*ts*^*/UAS-Chsy RNAi; UAS-mCD8::GFP/+* (or *MyoIA*^*ts*^*>Chsy RNAi*), shifting the culture temperature from 19°C to 30°C induces expression of the RNAi construct. As described above, CS was largely reduced when *Chsy RNAi* was induced by *MyoIA-Gal4* at an early developmental time point (2 days post egg laying) (Fig 2A-2Bii). In contrast, *MyoIA*^*ts*^*>Chsy RNAi* shifted to 30°C “late” (post eclosion) resulted in no reduction in anti-CS staining (S2D–S2Dii Fig). Furthermore, *MyoIA*^*ts*^*>Chsy RNAi* adults kept at 30°C for 20 days showed no reduction in CS staining (S2E–S2Eii Fig), indicating that CS is not actively replenished in the adult midgut. *MyoIA*^*ts*^*>Chsy RNAi* shifted to 30°C at the pupal stage showed intermediate anti-CS staining (S2F–S2Fii Fig). Rather than structured CS being present in the midgut, small and numerous puncta were observed in the midgut.

### *Chsy* is required for cell division control of ISCs during homeostasis

**Fig 3:**
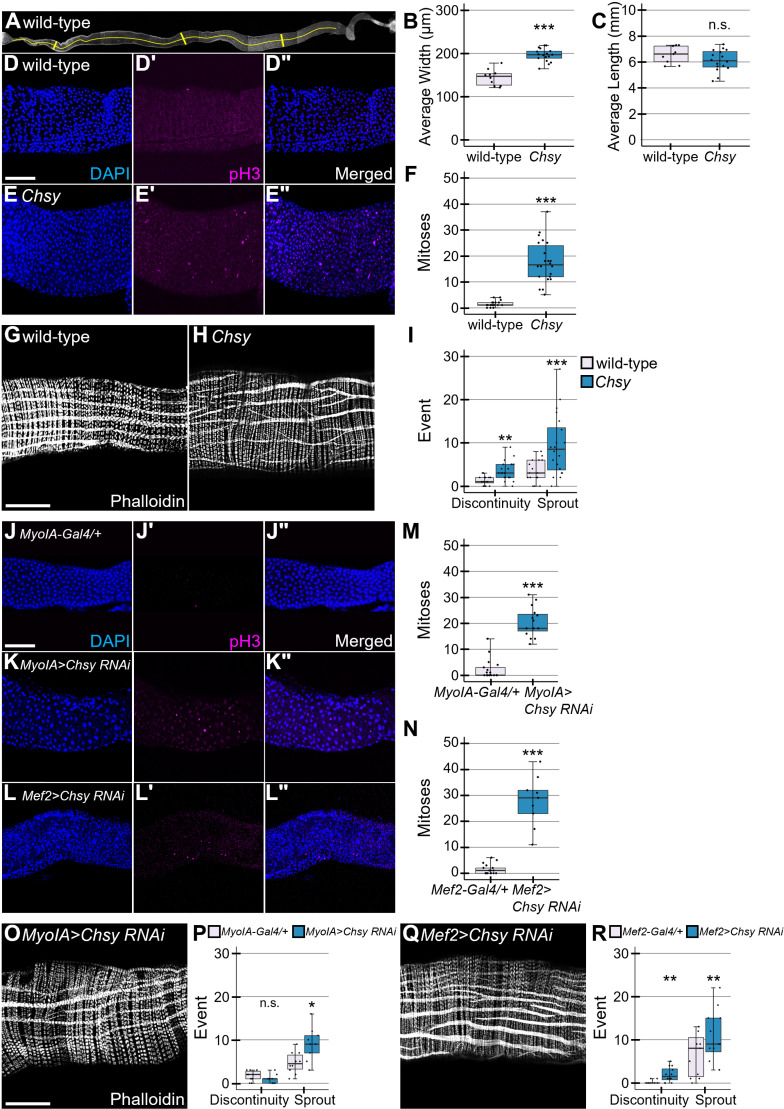
CS is necessary for midgut homeostasis. (A) Wild-type whole gut image. Midgut average width (B) was determined by averaging three widths from the anterior, middle, and posterior midgut as demonstrated by the vertical yellow lines (A). Midgut length (C) is demonstrated by the single horizontal line (A). Wild-type (D-D“) and *Chsy* (E-E”) midguts under homeostatic conditions were stained with DAPI (blue) and anti-pH3 antibody (magenta). (F) Quantification of pH3-positive cells in wild-type and *Chsy* mutant midguts. Wild-type (G) and *Chsy* (H) midguts stained with phalloidin to observe the longitudinal muscles (horizontal in representative images). (I) Quantification of longitudinal muscle defects in *Chsy* mutants. (J-R) Phenotypes of *Chsy* RNAi knockdown animals. Midguts from *MyoIA-Gal4/+* (J-J”), *MyoIA>Chsy RNAi* (K-K”), and *Mef2 > Chsy RNAi* (L-L”) animals were stained with DAPI (blue) and anti-pH3 antibody (magenta). Quantification of pH3-positive cells for *MyoIA>Chsy RNAi* (M) and *Mef2 > Chsy RNAi* (N). Phalloidin staining of *MyoIA>Chsy RNAi* (O) and *Mef2 > Chsy RNAi* (Q) midguts. Quantification of the muscle defects for *MyoIA>Chsy RNAi* (P) and *Mef2 > Chsy RNAi* (R). Boxes indicate the 25–75th percentiles, and the median is marked with a line. The whiskers extend to the highest and lowest values within 1.5 times the interquartile range. *P < 0.05; **P < 0.01; ***P < 0.001, n.s., not significant (two-sided, unpaired *t*-test). Scale bars: 100 μm.

To determine if the loss of CS affects midgut morphology, we quantified the length and thickness of the midgut samples from *Chsy* mutants as previously described [58]. The width was determined by averaging the widest part of the anterior, middle, and posterior midgut. Midgut length was defined as distance between the base of the cardia and the midgut-hindgut junction (Fig 3A). We found a significant increase in the midgut thickness in mutants while the gut length was not significantly affected (Fig 3B and 3C). The increased midgut width could result from several factors, including changes in epithelial cell shape, muscle layer thickness, and ISC proliferation rate. We quantified epithelial height and muscle layer thickness in wild-type and *Chsy* mutants and detected no significant difference between the genotypes (S3A and S3B Fig). Instead, the EC number is significantly increased in the *Chsy* posterior midgut compared to wild-type control (S3C Fig). Therefore, the midgut hyperplasia in *Chsy* mutants raised the possibility that CS plays a role in controlling ISC division during normal midgut homeostasis [58]. To measure ISC division, we analyzed mitotic activity of ISCs using anti-phosphohistone-H3 (pH3) antibody labeling. We found that the number of mitotic ISCs in the posterior midgut from *Chsy* is significantly increased compared to wild-type control (Fig 3D–3F). Taken together, the loss of *Chsy* resulted in elevated ISC mitotic activity during homeostasis, leading to an abnormally increased thickness of the midgut.

Our previous study showed that *Chsy* mutations disrupt the morphology and function of ovarian epithelial muscle sheath [48]. This is consistent with the idea that a putative *Drosophila* CSPG, Kon-tiki (Kon), is required for muscle development in embryos [59–61]and adults [62,63]. We, therefore, asked if *Chsy* affects visceral muscle structure.

Phalloidin staining for F-actin in a low magnification view revealed qualitative differences in musculature in *Chsy* mutants (Fig 3G and 3H). Using past research criteria for visceral muscle disruption phenotypes [64], we examined the longitudinal visceral muscle of *Chsy* mutant midguts at a higher magnification. Muscle discontinuity events were defined as when the main longitudinal muscles were non-continuous. Sprouting events were considered a continuous fiber that branched off from the main longitudinal muscles. We observed significant increases in muscle discontinuity and sprouting in comparison to wild-type (Fig 3I). The sprouting we observed was very similar to the ovary muscle phenotype in *Chsy* mutants, in which we described the similar phenomenon as muscle “branches” [48]. Taken together, we found that the longitudinal visceral muscle of *Chsy* mutant midguts is disrupted, with significant increases in muscle discontinuity and sprouting compared to wild-type.

Our finding that the ECs and visceral muscle are the major source of midgut CS suggest that locally synthesized CS is responsible for control of ISC proliferation and muscle morphology. To test this idea, we examined phenotypes of *Chsy RNAi* animals. We found that both *MyoIA>Chsy RNAi* and *Mef2 > Chsy RNAi* animals, which show a significant reduction in CS, recapitulate *Chsy* mutant phenotypes, including midgut hyperplasia (Fig 3J–3Lii), increased ISC division (Fig 3M and 3N), and muscle defects (Fig 3O–3R). Thus, these phenotypes are ascribed to the loss of local CS rather than systemic activity of *Chsy*.

### *Chsy* is required for downregulation of ISC proliferation at the regeneration termination

**Fig 4:**
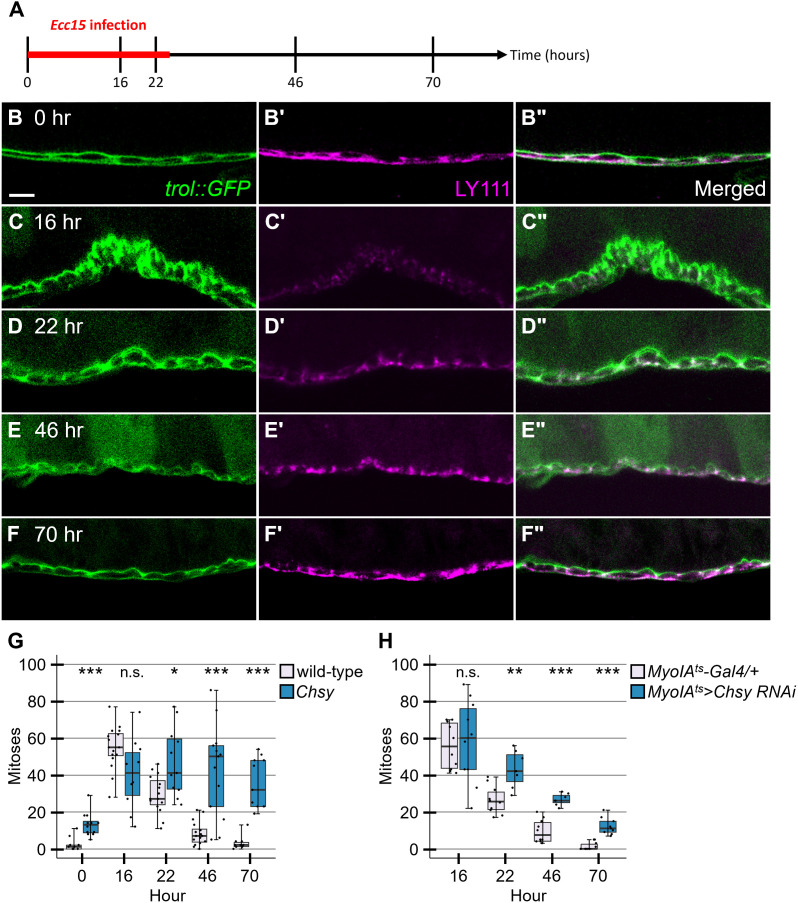
*Chsy* mutants fail to terminate regeneration. (A) Time course of regeneration. The flies were fed food with *Ecc15* for 24 hours as marked with the red bar. Midguts were dissected at 0, 16, 22, 46, and 70 hours after the beginning of *Ecc15* infection. (B-F“) CS dynamics during regeneration. Midguts form *trol::GFP* (green) flies under regeneration conditions were stained with LY111 (magenta) at indicated time points. (G) ISC mitotic activity in *Chsy* mutants during regeneration. The number of pH3-positive cells was quantified in wild-type and *Chsy* midguts throughout the time course of regeneration. (H) Regeneration assay for adult-specific *Chsy* knockdown using *MyoIA*^*ts*^*>Chsy RNAi*. Flies were shifted to 30°C to activate Gal4 one day before infection. Boxes indicate the 25–75th percentiles, and the median is marked with a line. The whiskers extend to the highest and lowest values within 1.5 times the interquartile range. *P < 0.05; **P < 0.01; ***P < 0.001; n.s., not significant (two-sided, unpaired t-test). Scale bar: 10 μm.

As CS is required for controlling ISC mitogenic activity during normal homeostasis, we asked if it is involved in damage-induced midgut regeneration. We first examined the dynamics of CS during regeneration (Fig 4A–4Fii). Upon feeding of gram-negative bacterium, *Ecc15*, the BM becomes thickened and massively disorganized with rounding of peristalsis muscle (16 hours after infection; Fig 4C), as reported previously in feeding of DSS [31] and *Pe* [43]. At this stage, LY111 staining is significantly decreased, and weak CS signals are observed as puncta (Fig 4Ci and 4Cii). During 22–46 hours post-infection initiation, the LY111 epitope gradually recovers as the BM morphology is being restored (Fig 4D–4Eii). At 70 hours, CS accumulation is observed as a mostly continuous signal in the BM (Fig 4F–4Fii), which is indistinguishable from the 0-hour control (Fig 4B–4Bii). Our data suggest that CSPGs in the BM appear to be degraded by tissue-damage and the process of regeneration termination is accompanied with the restoration of CS-containing BM.

We next asked if CS ablation affects ISC proliferation during regeneration. *Ecc15* infection substantially increases ISC division, as monitored by pH3 staining (Figs 4G and S4). The ISC mitotic activity reverts to a normal level within 70 hours after the beginning of infection (Figs 4G and S4), consistent with previous studies [39]. We found that *Chsy* mutant ISCs failed to properly downregulate mitotic activity at the end of regeneration (Figs 4G and S4). The number of pH3-positive cells at hours 22, 46, and 70 was significantly higher than control (Fig 4G). Thus, *Chsy* mutations prevented the cessation of ISC proliferation at the end of regeneration. Our results show that in addition to homeostasis, CS is required for proper regeneration termination. In this study, we use female flies for our experiments. However, we confirmed that *Chsy* mutant males show the same phenotypes as females regarding: (1) increased ISC proliferation during homeostasis, (2) muscle phenotypes (discontinuity and sprouting), and (3) failure of downregulation of ISC division at the regeneration termination stage (S5 Fig).

To determine if this effect of *Chsy* mutation on ISC proliferation can be ascribed to its expression of adult stages, we performed an adult-specific knockdown of *Chsy*. Toward this goal, we used the TARGET system, which employs a temperature-sensitive Gal80 (Gal80^ts^). We downregulated the *Chsy* gene in ECs using *MyoIA-Gal4 tubulin-GAL80*^*ts*^ (*MyoIA*^*ts*^) during regeneration [18]. Briefly, animals were raised at 19°C and shifted to 30°C at 24 hours before *Ecc15* infection to allow UAS transgene expression. We found that *MyoIA*^*ts*^*>Chsy RNAi* animals failed to slow down ISC division at the termination stage (Fig 4H). This result suggests that ISC mitotic activity is controlled by CS synthesized at adult stages, rather than accumulated abnormalities in the BM during development of *Chsy* mutants.

We next tested the effect of *Chsy* overexpression on ISC proliferation during regeneration. We used an EP line, *Chsy*^*EY11862*^ (referred as *Chsy-EP* in this paper), to overexpress *Chsy*. The EP lines bear a transgenic insertion which carries UAS binding sites for Gal4, and expression of the gene proximate to the insertion site can be induced by crossing a Gal4 driver. To induce *Chsy* overexpression in the ECs, *Chsy-EP* was crossed with *MyoIA-Gal4* (*MyoIA>Chsy-EP*). We found that *MyoIA>Chsy-EP* did not show any significant change in ISC mitotic activity during regeneration compared to control animals (*MyoIA-Gal4/+*) (S6A Fig). We confirmed that *Chsy* mRNA is actually overexpressed in *MyoIA>Chsy-EP* samples (S6B and S6C Fig). Our results show that *Chsy* is required, but not sufficient, to suppress ISC proliferation at the termination stage. We will discuss this phenomenon later.

### CS is required for barrier integrity

One mechanism of the CS’s requirement for normal regeneration termination is its possible involvement in shielding function of the midgut epithelium. Previous studies showed that a disruption of the septate junction abnormally upregulates ISC proliferation [65–70]. In addition, disruption of BM components, such as Viking (Type IV collagen), Laminin B1, and Peroxidasin (ColIV crosslinking enzyme), causes impaired barrier function and an inability to recover from regeneration [31]. Thus, the completion of the midgut barrier function is a prerequisite for the regeneration termination.

**Fig 5:**
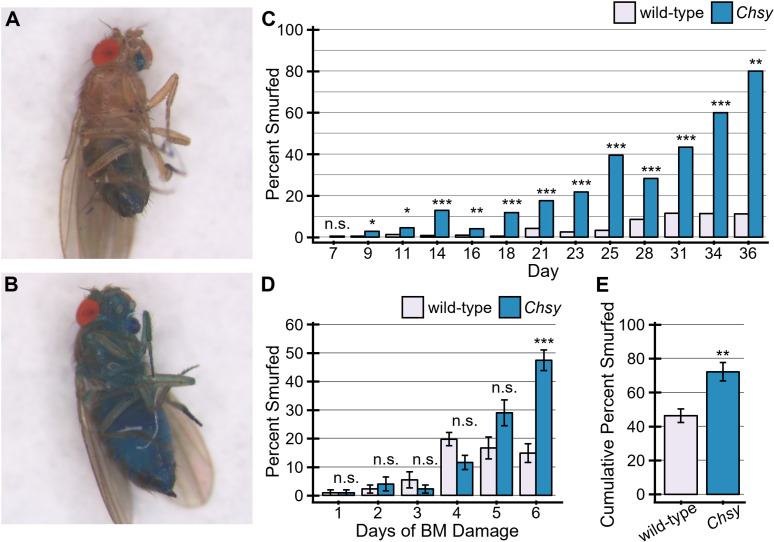
*Chsy* mutants display leaky guts. Representative images of normal (A) and smurfed (B) wild-type flies. (C) Quantification of aging smurf assay. *Chsy* mutants display significantly more smurfing throughout aging than wild-type flies. (D) Flies fed both blue dye and DSS to damage the BM. Resulting leakage was significantly higher for *Chsy* mutants on day 6. (E) Accumulated percentage of smurfed flies in wild-type and *Chsy*. Each value was calculated by summing the number of smurfed flies each day and dividing by the starting population. *P < 0.05; **P < 0.01; ***P < 0.001; n.s., not significant (Fisher’s exact test).

As CS depletion causes ISC hyperproliferation, we hypothesized that CS may be required for the barrier integrity. We quantified the epithelial barrier function by the established barrier integrity assay (Smurf assay) during the course of aging. Animals were fed normal food with the addition Blue Dye No. 1, and the loss of barrier function was determined when dye was observed outside the gut (Fig 5A and 5B). Percent smurfed was calculated by dividing new smurfs by the population number from the most recent observation time point. Both alive and dead smurfed flies were discarded to not interfere with the next observation time point. In wild-type, the barrier loss measured by the Smurf index gradually increases during aging (Fig 5C), as reported previously [71]. In *Chsy* mutants, this was accelerated. By day 9 (second observation time point), *Chsy* mutants had a significant difference in smurfing percentage. This statistically significant difference remained for the rest of observation time points (Fig 5C).

To test the effects of *Chsy* mutation on direct BM damage, flies were fed dextran sulfate sodium (DSS), which has been previously found to damage the BM [31], in addition to Blue Dye No. 1 for six days. We found that *Chsy* mutants had a significantly higher susceptibility to smurf by day 6 (Fig 5D) and a higher cumulative smurfed population (Fig 5E). Cumulative smurfed population was calculated by taking the sum of the number of smurfed flies from each day and dividing it by the starting population. Together, CS is essential for the barrier integrity in the midgut. There are three layers of physical barriers in midgut: the peritrophic membrane, epithelial cells sealed with SJs, and BM. We have previously observed that the *Chsy* mutation severely disrupts BM morphology in the ovary [48], suggesting that BM structural abnormality compromises barrier integrity in the midgut. However, future studies are needed to determine if the peritrophic membrane and/or epithelial layers are also affected by CS depletion.

We also examined the effect of *Chsy* overexpression on barrier integrity. A Smurf assay using *MyoIA>Chsy-EP* did not show any significant difference in the time course of the smurfed population compared to control animals (*MyoIA-Gal4/+*) (S6D Fig). Thus, similar to ISC division control, *Chsy* is required, but not sufficient, to sustain barrier integrity.

### Expression patterns of *Chsy* and *Chpf* during the regeneration

A recent study using single cell RNA-seq analysis identified CG43313 as the second most upregulated gene in ECs at the termination stage of regeneration induced by DSS feeding [44]. This gene encodes *Chondroitin polymerizing factor* (*Chpf*). In mammals, Chpf is a unique protein factor required for CS polymerization: it does not have an enzymatic activity, but it binds to Chsy [72]. The CS polymerization takes place by the action of the Chsy/Chpf complex, and both components are required for CS biosynthesis [72].

**Fig 6:**
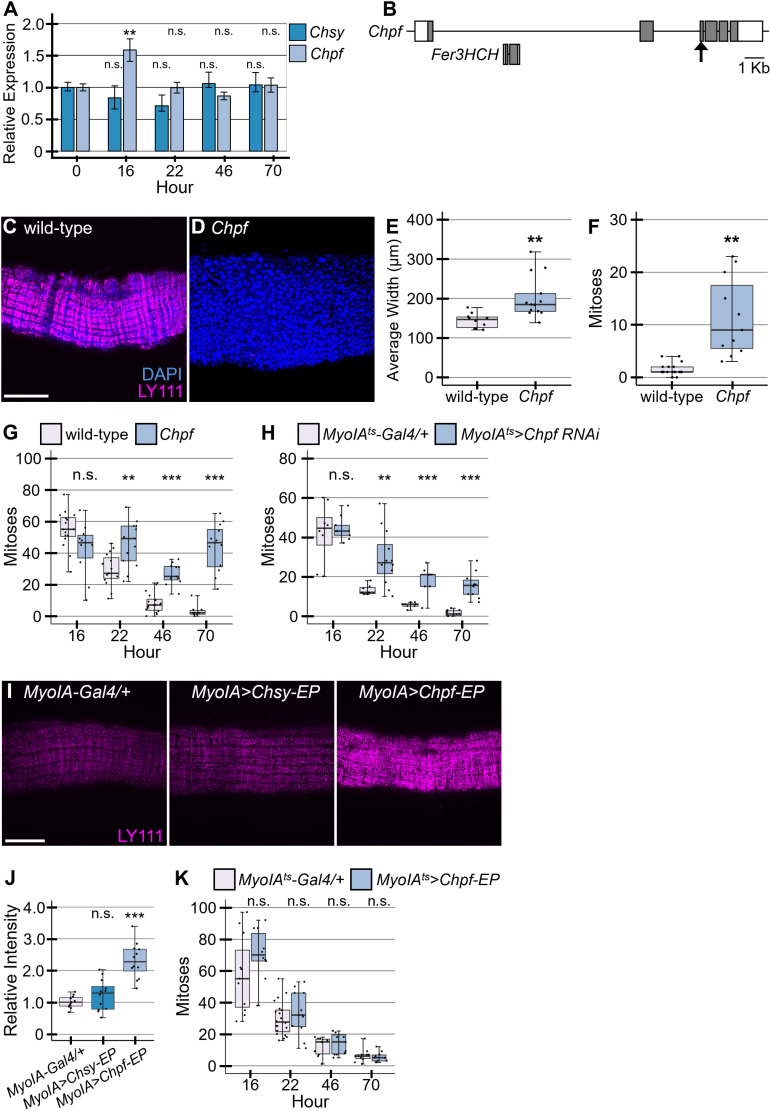
Chondroitin polymerizing factor regulates ISC proliferation. (A) RT-qPCR analysis of *Chsy* and *Chpf* expression patterns during regeneration in wild-type flies. The average expression levels of *Chsy* and *Chpf* at 0 hours post-infection were normalized to 1.0. (B) A schematic of the *Chpf* locus. The gene *Ferritin 3 heavy chain homologue* (*Fer3HCH*) exists within the first intron of the *Chpf* locus. Boxes and shaded areas show exons and the protein coding regions, respectively. The arrow indicates the sequence targeted by gRNA shown in S7 Fig. Wild-type (C) and *Chpf* (D) midguts were stained with DAPI (blue) and LY111 (magenta). (E) Quantification of midgut width is shown for wild-type and *Chpf* midguts. (F) Homeostasis pH3 quantification for *Chpf*. Number of pH3-positive cells counted as mitoses. (G) Regeneration assay for *Chpf*. (H) Regeneration assay for adult-specific *Chpf* knockdown using *MyoIA*^*ts*^*>Chpf RNAi*. Flies were shifted to 30°C to activate Gal4 one day before infection. (I) Guts were stained with LY111 for *MyoIA-Gal4/+* (left), *MyoIA>Chsy-EP* (middle), and *MyoIA>Chpf-EP* (right). (J) Quantification of LY111 staining with *Chpf* or *Chsy* overexpression in ECs. (K) *MyoIA*^*ts*^*>Chpf-EP* regeneration termination assay. Flies were shifted to 30°C to activate Gal4 one day before infection. All Boxes indicate the 25–75th percentiles, and the median is marked with a line. The whiskers extend to the highest and lowest values within 1.5 times the interquartile range. *P < 0.05; **P < 0.01; ***P < 0.001; n.s., not significant (two-sided, unpaired *t*-test). Scale bars: 100 μm.

Bacteria feeding and DSS treatment induce different midgut responses or trigger similar phenomena at different timing after the induction [27,30]. To determine if bacterial infection induces *Chpf*, we tested expression patterns of *Chsy* and *Chpf* during the regeneration induced by *Ecc15* feeding by RT-qPCR. We found that *Ecc15* infection induces a modest, but statistically significant peak of *Chpf* expression at 16 hours after feeding (Fig 6A). This induction occurred earlier than its induction by DSS treatment [44]. We did not observe any significant changes of *Chsy* expression during this time-course (Fig 6A).

### Roles of Chondroitin polymerizing factor in CS biosynthesis

To examine the role of *Chpf* in midgut regeneration, we isolated a *Chpf* mutant allele by CRISPR/Cas9 mutagenesis (Fig 6B). As there is an intronic gene within the first intron of the *Chpf* locus (*Ferritin 3 heavy chain homologue*, *Fer3HCH*), we designed a gRNA to target the protein coding sequence in the third exon (arrow). The CRISPR/Cas9-mediated mutagenesis induced a deletion of 11 base pairs, which caused a frame shift, resulting in a truncated protein (S7 Fig). This allele, which was referred to as *Chpf*^*424*^, encodes the first 294 amino acid residues of the wild-type protein (819 amino acids), lacking important protein domains. LY111 staining revealed that CS is undetectable in the *Chpf*^*424*^ midgut, similar to *Chsy*, showing that *Drosophila Chpf* is also essential for CS biosynthesis (Fig 6C and 6D). We also found that ISC division is abnormally increased during homeostasis in *Chpf* mutants, resulting in a thickened midgut (Fig 6E and 6F). Additionally, *Chpf* mutants had significantly more proliferation during regeneration at hours 22, 46, and 70 after damage initiation, thus failing to properly terminate regeneration (Fig 6G). We found that *Chpf* mutant samples show a higher level of variability of pH3-positive cells among individuals compared to wild-type. Although there was a slight drop of 46-hour *Chpf* mutant samples compared to 22- and 70-hour samples, it is unlikely that there is a specific, biological cause for it. Our observations confirmed the function of CS in ISC control and demonstrated non-redundant functions of *Chsy* and *Chpf* in CS biosynthesis.

Adult-specific *Chpf* knockdown experiments demonstrated that *MyoIA*^*ts*^*>Chpf RNAi* failed to downregulate ISC proliferation at later stages of regeneration (Fig 6H). This further supports the idea that CS Biosynthesis during regeneration is required for proper control of ISC division.

To determine if *Chpf* overexpression alone increases the CS biosynthesis, we overexpressed *Chpf* or *Chsy* using EP lines. In addition to *Chsy-EP* used above, we used *Chpf*^*EY00553*^ (referred as *Chpf-EP* in this paper) for *Chpf* overexpression. *Chpf-EP* and *Chsy-EP* were crossed to *MyoIA-Gal4*, and CS in the midgut was quantified. LY111 staining of *MyoIA>Chpf-EP* midguts showed a significantly increased level of CS (Fig 6I and 6J). On the other hand, no detectable change was observed in *MyoIA>Chsy-EP* midguts. Thus, overexpression of *Chpf*, but not *Chsy*, stimulates increased CS biosynthesis, suggesting that Chpf is a rate-limiting component of the CS biosynthetic machinery. This is consistent with a recent observation that a gain-of-function allele of *mig-22*, the *C. elegans* orthologue of *Chpf*, promotes CS biosynthesis [73]. Our results support the idea that *Chpf*, which is induced by tissue damage during regeneration, upregulates the CS biosynthesis to trigger the CS remodeling and stimulate tissue repair.

We found that *Chpf* overexpression in the entrocytes by *MyoIA*^*ts*^ one day prior to infection does not slow proliferation at the beginning of regeneration (Fig 6K). Additionally, the overall regeneration curve appears normal for *MyoIA*^*ts*^*>Chpf-EP* flies. Therefore, although CS is required for the downregulation of ISC mitotic activity, overexpression of *Chpf* at the initiation stage is not sufficient to halt ISC division. This suggests that CS plays a permissive role, rather than an instructive signal, during the termination process.

## Discussion

Compared to the induction of ISC proliferation, the mechanism of regeneration termination is understudied. Since dysregulation of this process results in a high risk of cancer [74,75], it is important to understand how the tissue recognizes when regeneration is completed and properly downregulates mitotic pathways. Our study showed that CS is required for ISC control during both normal homeostasis and regeneration as well as for midgut barrier integrity. CS polymerization in *Drosophila* requires two genes, *Chsy* and *Chpf*.

We found that CS repair is necessary for downregulating ISC mitotic activity, but a higher level of CS does not inhibit ISC division. Given that all other BM components are still being restored at the beginning and in the middle of regeneration, increased CS alone is unlikely to complete the BM repair. On the other hand, the induction of *Chpf* by tissue damage and its ability to enhance CS production suggest that increased CS production may facilitate the damage-repair at the end of regeneration. Thus, our results indicate that CS acts as a permissive signal. In this respect, the role of Chsy and Chpf is distinct from that of Sulf1 and Mov10, two genes we have previously identified as a “break” of ISC division [41,43] (discussed below). These ISC “breaks” inhibit ISC division when prematurely misexpressed. The identification of the two distinct classes of molecules suggest that regeneration termination occurs through two steps.

In the first step, specific conditions, or prerequisites, are checked to confirm that damage repair has been completed and the regeneration process can be terminated. Based on our observations, we propose that CS remodeling, one of the termination hallmarks, acts as a prerequisite to proceed to the next step. Once specific prerequisites are satisfied, a few mechanisms are known to act to downregulate ISC proliferation back to homeostasis state. For example, Dpp negatively controls ISC division during homeostasis and regeneration [37,76]. In addition, a structural modification of co-receptors for growth factors occurs at the end of regeneration [41]. Many of the mitotic ligands that stimulate ISC proliferation are heparan sulfate (HS)-dependent factors and use HS proteoglycans as a co-receptor. De-sulfation of HS chains by the extracellular sulfatase Sulf1 removes ligand-binding sites on HS, leading to cessation of mitogen signaling at the termination stage [41]. Furthermore, a microRNA-mediated network provides an additional layer of ISC activity control by influencing the transcript levels of ISC regulators [43]. Additionally, following a recovery period after gut damage, a current of calcium promotes enterocyte maturation and subsequently ISC downregulation [44].

It is interesting to compare the role of CS in ISC control with that of septate junctions (SJs), functional counterparts of vertebrate tight junctions. SJs are the structural basis for the epithelial barrier function and directly affect ISC proliferation. Depletion of SJ components results in increased ISC proliferation, the accumulation of morphologically abnormal ECs, and impaired barrier integrity [65–71]. Thus, both SJs and CS are critical to prevent the leakage of the midgut epithelium and link between the epithelial integrity and ISC activity control. Importantly, a molecular mechanism by which SJ repair represses ISC division at the termination stage has been proposed [69]. When ISCs differentiate toward ECs following tissue damage, a SJ protein Tsp2A is produced. In addition to its function as a SJ component, Tsp2A is actively internalized from the SJs and mediates the degradation of atypical protein kinase C (aPKC). aPKC is a cell polarity regulator and is known to antagonize Hippo signaling. Therefore, the restoration of Tsp2A at the SJ turns off Yorkie (Yki), a transcriptional coactivator, and Jak/Stat signaling downstream of Yki. The link between SJ and Yki-dependent ISC proliferation was also reported for two other SJ components, Snakeskin and Mesh [70], further supporting the idea that the completion of the SJ repair acts as a prerequisite in the first step of regeneration termination. Molecular mechanisms for sensing the repair of CS-containing BM remain to be elucidated.

The BM is known to function as signaling platforms in addition to its roles as a structural basis for cellular substrates. For example, perlecan, a heparan sulfate proteoglycan, affects cell signaling by sequestering multiple growth factor ligands [51]. To examine if mitogenic pathways are altered by loss of CS, we performed RT-qPCR using midgut samples from wild-type and *Chsy* mutant flies. We found that proliferative pathways were overall slightly upregulated for *Chsy* mutants as *upd3*, *socs36e, dpp, hh, wg, and vn* were all found to have higher expression than wild-type (S8 Fig). However, only *hh* was significantly upregulated (*P* < 0.05). Therefore, it is possible that upregulation of mitogenic signaling contributes in some extent to the increased ISC division in *Chsy* mutants.

What CSPG core-protein genes are responsible for the *Chsy/Chpf* midgut phenotypes remains to be determined. One candidate molecule is Wdp [54]. Wdp regulates ISC division through a negative feedback loop of the Jak/Stat pathway. Later, Wdp was found to be a CSPG [77]. The muscle phenotypes of *Chsy* mutants may be related to Kon. Kon, a well-established muscle regulator, encodes the *Drosophila* orthologue of mammalian NG2/CSPG4 and thus, it is a putative CSPG [59–63]. Although it is unknown whether Kon plays a role in the midgut, the *Chsy* muscle phenotypes suggest that a similar CSPG-dependent mechanism may regulate visceral muscle development. Further studies are needed to elucidate how CS/CSPGs organize midgut homeostasis and regeneration through chemical and mechanical signaling.

In vertebrates, developmental roles of CS in the skeletal system have been well established. Chsy-1 mutations in humans result in Temtamy preaxial brachydactyly, a disease characterized by morphological abnormalities, including limb malformations, facial dysmorphism, and short stature [78,79]. On the other hand, CS is an evolutionarily old glycosaminoglycan and shared by primitive species with no bone and cartilage, indicating that CS’s original role was not in skeletal development. CS-deficient animal models in *C. elegans* and *Drosophila* will be important genetic tools to elucidate the original functions of CS.

## Materials and methods

### Fly stocks and husbandry

Oregon-R and *w*^*1118*^ were used as wild-type stocks. Additional fly strains used were *Chsy*^*2*^ [48], *Trol::GFP* (DGRC #110807), *MyoIA-Gal4*^*NP0001*^ (Kyoto DGGR #112001), *esg-Gal4*^*NP6267*^ (Kyoto DGGR #113886), *Mef2-Gal4* (BDSC #27390), *Lpp-Gal4* (BDSC # 84297), *tub-Gal80*^*ts*^ (BDSC #7108), *UAS-Chsy RNAi* (VDRC #29084), *UAS-Chpf RNAi* (VDRC #26519), *Chpf*^*EY00553*^ (BDSC #16537), *Chsy*^*EY11862*^ (BDSC #20705), and *UAS-GFP* (BDSC #1521). All flies used in the experiments were 4–7-day old females unless otherwise noted. Flies were raised at 25°C on standard cornmeal fly medium unless otherwise noted. Detailed genotypes used in individual experiments are listed in S1 Table.

*Chpf*^*424*^ mutant strain was generated by CRISPR/Cas9-mediated nonhomologous end joining as previously described [48,77]. A sgRNA sequence targeting *Chpf*, chosen using CRISPR Optimal Target Finder, was cloned into pU6-BbsI-chiRNA (a gift from Melissa Harrison, Kate O’Connor-Giles, and Jill Wildonger). The sgRNA-containing plasmid was injected into the *y w; nos-Cas9(y+)/CyO* strain by Genetivision Corp. to generate small deletions in the *Chpf* gene. Resultant deletions were screened via PCR, verified by Sanger sequencing, followed by backcrossing with Oregon-R strain for five generations. The sgRNA sequence used is described in S7 Fig.

Fly stocks were reared on a standard cornmeal fly medium at 25°C except for those containing tub-GAL80^ts^, which prevents Gal4-mediated UAS transgene expression at 19°C but activates it at 30°C [57]. For the source of CS experiment, 3-day old females were used. In Fig 2A-2Bii, *MyoIA-Gal4*^*ts*^ crosses were shifted to 30°C to drive expression 2 days post egg laying and raised at 30°C continuously until dissection, whereas “late” crosses (S2D–S2Eii Fig) were kept at 19°C and placed in a 30°C incubator following eclosion.

## Immunohistochemistry

Female midguts were dissected in PBS and placed in fixative solution (3.7% formaldehyde in PBS) for 1 hour. They were subsequently washed 3 times for 10 minutes with PBST (PBS with 0.1% Triton X-100). The midguts were then incubated at room temperature for 1 hour in blocking solution (PBS with 10% NGS). Following blocking, the samples were incubated with primary antibody at 4°C overnight. The next day, they were washed 3 times with PBST and placed in secondary antibody solution for either 2 hours at room temperature or overnight at 4°C. After the secondary antibody staining, the midguts were washed 3 more times with PBST and stained with DAPI for 10 minutes. One final wash with PBST was completed, and then the samples were mounted with VECTASHIELD Antifade Mounting Medium (H-1000, Vector Laboratories, Burlingame, CA). For wing disc staining, the same staining protocol was followed except that the discs were fixed for 30 minutes instead of 1 hour.

Primary antibodies used were rabbit anti-pH3 (1:1000, 06–570, Millipore, Darmstadt, Germany) and mouse anti-CS-A (1:100, Tokyo Chemical Industry, LY111). Secondary antibodies used were conjugated with Alexa Fluor 488, 568, or 633 (1:500, Thermo Fischer Scientific). To observe muscle morphology, F-actin was stained with phalloidin (1:250, Thermo Fisher Scientific, A22284). Images were acquired on a LSM710 (Carl Zeiss, Oberkochen, Germany) confocal microscope and processed with FIJI.

### Midgut width and length measurements

Measuring midgut dimensions has been previously described [58]. Briefly, a single image of the midgut was created from tiled images using 10x magnification with Zen imaging software (Zeiss). The width was determined by averaging the widest part of the anterior, middle, and posterior midgut. Midgut length was determined by drawing a line from the base of the cardia to the midgut-hindgut junction. Midgut width and length were measured using FIJI.

### Muscle phenotype quantification

Visceral longitudinal muscle phenotype events were quantified from a single 20x image of the posterior midgut on a LSM710 (Carl Zeiss, Oberkochen, Germany) confocal microscope. The events were defined as previously described [64]. Briefly, discontinuity events occurred when the main longitudinal muscles were non-continuous. Sprout events were considered a continuous fiber that branched off from the main longitudinal muscles.

### Barrier integrity assay

Barrier integrity assay, or so-called Smurf assay, was adapted from Rera *et al.* 2012 [80]. Fresh food was prepared with 2% blue dye (FD&C Blue #1, Spectrum Chemical, FD110). 15–20 four-day old flies were placed into individual blue food vials and transferred to a 30°C incubator. Starting at day seven, the flies were check every two to three days for smurfing and/or death. At each check point, the flies were transferred to fresh blue food. Smurfing was indicated by the fly turning blue outside of the digestive tract. Flies with extensive blue outside of their digestive tract and throughout their hemolymph were considered smurfs. ‘Light smurfs’ [81] were not included in the smurf count in this paper. Percent smurfed was calculated by taking the new number of smurfed flies and dividing it by the prior population number. Cumulative smurf percent was calculated by dividing the sum of all smurfs from each day by the original starting population number. Both alive and dead smurfed flies were included in the percent smurfed and cumulative smurf percentages.

DSS (dextran sulfate sodium salt colitis grade, MP Biomedicals, CAS number 9011-18-1; 36,0000–50,000 MW) was used to assess the barrier integrity in response to basement membrane damage. As previously described [31], 250 µl of a 5% sucrose solution containing 2% blue dye and 3% DSS was added directly to Whatman filter paper in an empty vial. The control vials contained no DSS. Wild-type and *Chsy* flies were added to separate vials. Every 24 hours, the flies were placed in new vials containing freshly soaked Whatman filter paper. In addition, every 24 hours the flies were checked for smurfing.

### Induction of regeneration

A gram-negative bacterium, *Erwinia carotovora* strain 15 (*Ecc15*, a gift from Nicholas Buchon and Aiguo Tian) was used to induce midgut regeneration [27]. A culture of *Ecc15* was grown overnight. To induce feeding of the bacteria, 4–6-day old flies were placed in empty vials two hours prior to infection. Then, the flies were placed in vials containing Whatman filter paper soaked in 250 µl of a 2.5% sucrose containing OD_600_ = 100 *Ecc15* or 250 µl 5% sucrose (control). The flies were placed back in normal food vials after 24 hours. Dissection time points were 16, 22, 46, and 70 hours after the start of infection.

For overexpression of *Chsy* and *Chpf*, *Chsy*^*EY11862*^ (*Chsy-EP*) and *Chpf*^*EY00553*^ (*Chpf-EP*) were driven by *MyoIA-Gal4*^*ts*^. The crosses and progeny were kept at 19°C. One day prior to infection, the progeny was shifted to 30°C and kept at that temperature throughout regeneration. For control, *MyoIA-Gal4*^*ts*^ flies were crossed with wild-type.

For adult-specific knockdown of *Chsy* or *Chpf*, *UAS-Chsy RNAi* or *UAS-Chpf RNAi* was driven by *MyoIA-Gal4*^*ts*^. One day prior to infection, the progeny was shifted to 30°C to induce expression of RNAi transgenes. For control, *MyoIA-Gal4*^*ts*^ flies were crossed with wild-type.

### RT-qPCR

Total RNA from 20 5-day old female midguts was extracted using TRIzol (15596026, Thermo Fisher Scientific), treated with RNase-Free DNase I (79524, Qiagen), and purified using a Direct-zol RNA MiniPrep kit (R2050, Zymo Research). A SuperScript III First-Strand Synthesis SuperMix kit (18080–400, Thermo Fisher Scientific) was used to synthesize the purified RNA to cDNA. Using LightCycler 480 SYBR Green I Master mix (04707516001, Roche Diagnostics) in a LightCycler 480 Instrument II (Roche, Basel, Switzerland), qPCR assays were run on triplicate for each of the four independent biological replicates. Fold changes were calculated using the ΔΔCt method, and Act5C expression was used for normalization. Primers used were based on previous studies [35,41,82] or chosen from FlyPrimerBank [83]. Primer sequences are listed in S2 Table.

### Statistical Analyses

The Fischer’s exact test was obtained for p-values for the smurf assay using R (http://cran.r-project.org/). Unpaired, two-sided t-tests were used for all other statistical significance tests using JMP (https://www.jmp.com/en_us/home.html).

## Supporting information

S1 Table Genotypes of *Drosophila* strains. (PDF)

S2 Table Primers used in RT-qPCR experiments. (PDF)

S1 Fig The localization of CS in the midgut. (PDF)

S2 Fig The source of CS in the midgut BM. (PDF)

S3 Fig The midgut epithelial height, muscle layer thickness, and the number of ECs in *Chsy* mutant. (PDF)

S4 Fig ISC proliferation in *Chsy* mutants during regeneration. (PDF)

S5 Fig *Chsy* mutant males show the consistent phenotypes with females. (PDF)

S6 Fig The effects of *Chsy* overexpression in the midgut. (PDF)

S7 Fig Generation of *Chpf*^*424*^ allele. (PDF)

S8 Fig Mitogenic signaling is upregulated in the *Chsy* mutants. (PDF)

S1 Data Fig 1 Data. (XLSX)

S2 Data Fig 2 Data. (XLSX)

S3 Data Fig 3 Data. (XLSX)

S4 Data Fig 4 Data. (XLSX)

S5 Data Fig 5 Data. (XLSX)

S6 Data Fig 6 Data. (XLSX)

S7 Data S3 Fig Data. (XLSX)

S8 Data S5 Fig Data. (XLSX)

S9 Data S6 Fig Data. (XLSX)

S10 Data S8 Fig Data. (XLSX)
