# Supplementary material for: Chondroitin sulfate regulates proliferation of Drosophila intestinal stem cells
Source: PLoS Genet. 2025 May 9;21(5):e1011686. doi: 10.1371/journal.pgen.1011686 (PMC12063844; doi:10.1371/journal.pgen.1011686)
Supplement: S1 Table — (PDF) [file pgen.1011686.s001.pdf]

| Figure | Genotype |
| --- | --- |
| Fig 1A, 1B, 1H | *Oregon-R* |
| Fig 1C-1E, 1G | *trol::GFP* |
| Fig 1I | *$Chsy^{2}$* |
| Fig 2A, 2E | *MyoIA-Gal4 tub-$Gal80^{ts}$/+* |
| Fig 2B, 2E | *MyoIA-Gal4 tub- $Gal80^{ts}$/+; UAS-Chsy RNAi/UAS-mCD8::GFP* |
| Fig 2C, 2F | *Mef2-Gal4/+* |
| Fig 2D, 2F | *Mef2-Gal4/UAS-Chsy RNAi* |
| Fig 3A-3D, 3F | *$w^{1118}$* |
| Fig 3B, 3C, 3E, 3F, 3H, 3I | *$Chsy^{2}$* |
| Fig 3G, 3I | *Oregon-R* |
| Fig 3J, 3M, 3P | *MyoIA-Gal4 tub-$Gal80^{ts}$/+* |
| Fig 3K, 3M, 3O, 3P | *MyoIA-Gal4 tub-$Gal80^{ts}$/+; UAS-Chsy RNAi/UAS-mCD8::GFP* |
| Fig 3L, 3N, 3Q, 3R | *Mef2-Gal4/UAS-Chsy RNAi* |
| Fig 3N, 3R | *Mef2-Gal4/+* |
| Fig 4B-4F | *trol::GFP* |
| Fig 4G | *$w^{1118}$* |
| Fig 4G | *$Chsy^{2}$* |
| Fig 4H | *MyoIA-Gal4 tub-$Gal80^{ts}$/+* |
| Fig 4H | *MyoIA-Gal4 tub-$Gal80^{ts}$/+; UAS-Chsy RNAi/+* |
| Fig 5A-5E | *Oregon-R* |
| Fig 5C-5E | *$Chsy^{2}$* |
| Fig 6A, 6C | *Oregon-R* |
| Fig 6D-6G | *$Chpf^{424}$* |
| Fig 6E-6G | *$w^{1118}$* |
| Fig 6H-6K | *MyoIA-Gal4 tub-$Gal80^{ts}$/+* |
| Fig 6H | *MyoIA-Gal4 tub-$Gal80^{ts}$/+; UAS-Chpf RNAi/+* |
| Fig 6I, 6J | *$Chsy^{EY11862}$/+; MyoIA-Gal4 tub-$Gal80^{ts}$/+* |
| Fig 6I-6K | *$Chpf^{EY00553}$/+; MyoIA-Gal4 tub-$Gal80^{ts}$/+* |
| S1 Fig | *MyoIA-Gal4 tub-$Gal80^{ts}$/UAS-mCD8::GFP* |
| S2A Fig | *esg-Gal4 UAS-GFP/+; tub-$Gal80^{ts}$/UAS-Chsy RNAi* |
| S2B Fig | *Lpp-Gal4/UAS-Chsy RNAi* |
| S2C Fig | *tub-$Gal80^{ts}$/+; pros-Gal4/ UAS-Chsy RNAi* |
| S2D-S2F Fig | *MyoIA-Gal4 tub-$Gal80^{ts}$/+; UAS-Chsy RNAi/UAS-mCD8::GFP* |
| S2G Fig | *UAS-Chsy RNAi* |
| S3A-S3C Fig | *Oregon-R* |
| S3A-S3C Fig | *$Chsy^{2}$* |
| S4 Fig | *$w^{1118}$* |
| S4 Fig | *$Chsy^{2}$* |
| S5A-S5C Fig | *Oregon-R* |
| S5A-S5C Fig | *$Chsy^{2}$* |
| S6A-S6D Fig | *MyoIA-Gal4 tub-$Gal80^{ts}$/+* |
| S6A-S6D Fig | *$Chsy^{EY11862}$/+; MyoIA-Gal4 tub-$Gal80^{ts}$/+* |
| S8 Fig | *Oregon-R* |
| S8 Fig | *$Chsy^{2}$* |

**S1 Table. Genotypes of *Drosophila* strains**
