# Supplementary material for: Chondroitin sulfate regulates proliferation of Drosophila intestinal stem cells
Source: PLoS Genet. 2025 May 9;21(5):e1011686. doi: 10.1371/journal.pgen.1011686 (PMC12063844; doi:10.1371/journal.pgen.1011686)
Supplement: S2 Table — (PDF) [file pgen.1011686.s002.pdf]

**Gene**		**Sequence**	**FlyPrimerBank/ References**
Act5C	forward	5'-GGCGCAGAGCAAGCGTGGTA-3'	[82]
	reverse	5'-GGGTGCCACACGCAGCTCAT-3'	
upd3	forward	5'-TACGCATCTGGACTGGGAGA-3'	[41]
	reverse	5'-TACGCATCTGGACTGGGAGA-3'	
socs36e	forward	5'-ATGGGTCATCACCTTAGCAAGT-3'	PP11279
	reverse	5'-TCCAGGCTGATCGTCTCTACT-3'	
dpp	forward	5'-TGGCGACTTTTCAAACGATTGT-3'	PP5962
	reverse	5'-CAGCGGAATATGAGCGGCAA-3'	
hh	forward	5'-GCAAACGATGCGCCACATT-3'	PP25933
	reverse	5'-CGCCCTATGACGACCCAATC-3'	
wg	forward	5'-CCAACCCCACGAAGTACAGA-3'	[35]
	reverse	5'-CATGGATGGGGTGGTTTAAG-3'	
vn	forward	5'-GAACGCAGAGGTCACGAAGA-3'	PD80001
	reverse	5'-GAGCGCACTATTAGCTCGGA-3'	
Chsy	forward	5'-CCGGGAACTGCTCTACGGATACC-3'	designed in this study
	reverse	5'-GGCTGCCCAGGAGACTTTGC-3'	
Chpf	forward	5'-CTACTACTCCAAGCACCATCTG-3'	designed in this study
	reverse	5'-GAAAGTTGTGCGTCCCATTG-3'	

S2 Table. Primers used in RT-qPCR experiments
