# Supplementary material for: Chondroitin sulfate regulates proliferation of Drosophila intestinal stem cells
Source: PLoS Genet. 2025 May 9;21(5):e1011686. doi: 10.1371/journal.pgen.1011686 (PMC12063844; doi:10.1371/journal.pgen.1011686)
Supplement: S1 Fig — (PDF) [file pgen.1011686.s003.pdf]

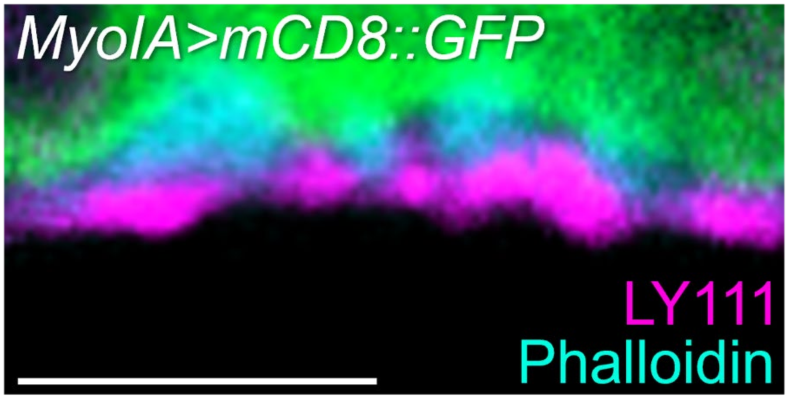

S1 Fig. The localization of CS in the midgut.
LY111 staining of *MyoIA>mCD8::GFP*. Midguts were stained with LY111 (magenta) and phalloidin (cyan). Peristalsis muscles are marked by phalloidin. LY111 staining did not show significant overlap with the basal membrane of the ECs (green). Scale bar: 5 µm.
