# Supplementary material for: Chondroitin sulfate regulates proliferation of Drosophila intestinal stem cells
Source: PLoS Genet. 2025 May 9;21(5):e1011686. doi: 10.1371/journal.pgen.1011686 (PMC12063844; doi:10.1371/journal.pgen.1011686)
Supplement: S2 Fig — (PDF) [file pgen.1011686.s004.pdf]

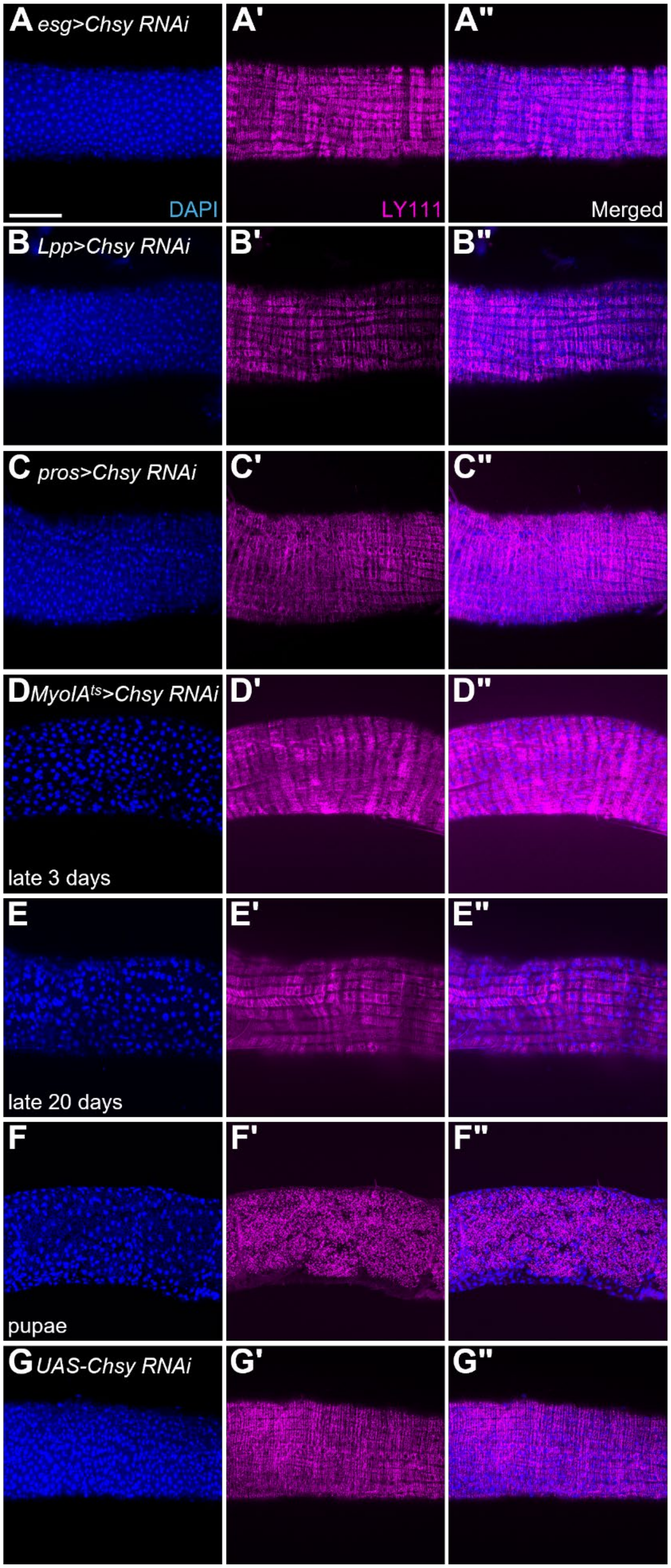

A esg>Chsy RNAi
A'
A"
DAPI
LY111
Merged
B Lpp>Chsy RNAi
B'
B"
C pros>Chsy RNAi
C'
C"
D MyoIA^ts>Chsy RNAi
D'
D"
late 3 days
E
E'
E"
late 20 days
F
F'
F"
pupae
G UAS-Chsy RNAi
G'
G"

S2 Fig. The source of CS in the midgut BM.
Midguts were stained with DAPI (blue) and LY111 (magenta) to investigate the source of CS in the midgut BM. Different Gal4 drivers were crossed with *UAS-Chsy RNAi* to knockdown CS production from specific cell/tissue groups: *esg-Gal4* (A-A") drives *UAS-Chsy RNAi* in ISCs, *Lpp-Gal4* (B-B") in fat body, *pros-Gal4* (C-C") in EEs, and *MyoIA^{ts}-Gal4* (D-F") in ECs. *UAS-Chsy RNAi/+* was used as a control (G-G"). *MyoIA^{ts}-Gal4* flies were under temperature control. "Late" induction flies were shifted to 30°C after eclosion to drive *UAS-Chsy RNAi* expression for either 3 (D-D") or 20 (E-E") days. Continued expression of *UAS-Chsy RNAi* over 20 days does not lead to a reduction of CS in the midgut. (F- F") Animals were shifted to drive expression during the early pupal stage. LY111 staining revealed disorganized CS staining. Scale bar: 100 µm.
