# Supplementary material for: Chondroitin sulfate regulates proliferation of Drosophila intestinal stem cells
Source: PLoS Genet. 2025 May 9;21(5):e1011686. doi: 10.1371/journal.pgen.1011686 (PMC12063844; doi:10.1371/journal.pgen.1011686)
Supplement: S3 Fig — (PDF) [file pgen.1011686.s005.pdf]

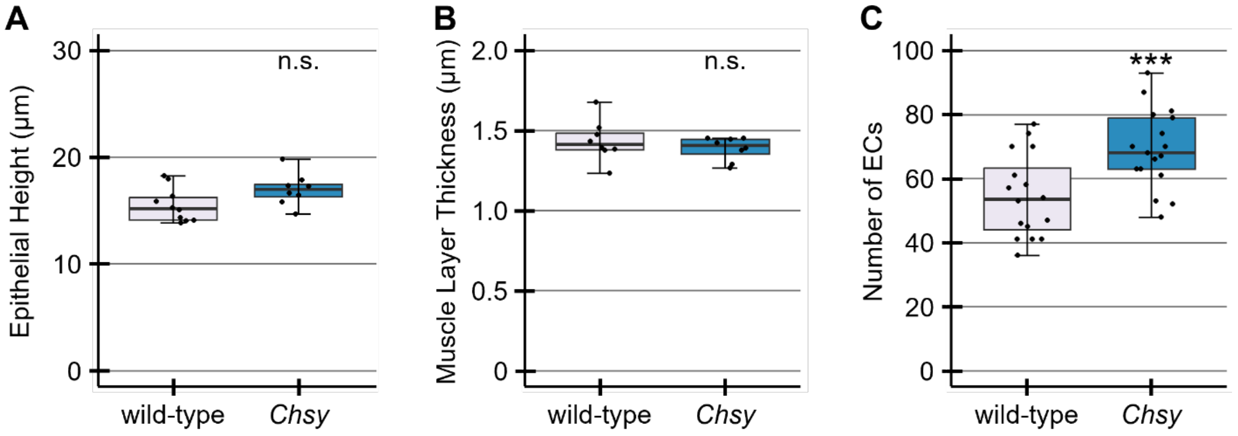

S3 Fig. The midgut epithelial height, muscle layer thickness, and the number of ECs in *Chsy* mutant.
Quantification of midgut epithelial height (A), muscle layer thickness (B), and the number of ECs (C) in wild-type and *Chsy* mutants. (A) The average height of the midgut epithelial cells was determined by measuring five cells from each gut (wild-type: n=10, *Chsy*: n=8). No significant difference was observed between the genotypes. (B) The average thickness of the muscle layer was calculated by averaging five locations of each gut (wild-type: n=8, *Chsy*: n=8). No significant difference was observed between the genotypes. (C) The number of the ECs in a specific area of the posterior midgut was counted (wild-type: n=16, *Chsy*: n=17). The number of ECs per given area was significantly increased in *Chsy* mutants. Boxes indicate the 25-75th percentiles, and the median is marked with a line. The whiskers extend to the highest and lowest values within 1.5 times the interquartile range. ***$P<0.001$; n.s., not significant (two-sided, unpaired *t*-test).
