# Supplementary material for: Chondroitin sulfate regulates proliferation of Drosophila intestinal stem cells
Source: PLoS Genet. 2025 May 9;21(5):e1011686. doi: 10.1371/journal.pgen.1011686 (PMC12063844; doi:10.1371/journal.pgen.1011686)
Supplement: S4 Fig — (PDF) [file pgen.1011686.s006.pdf]

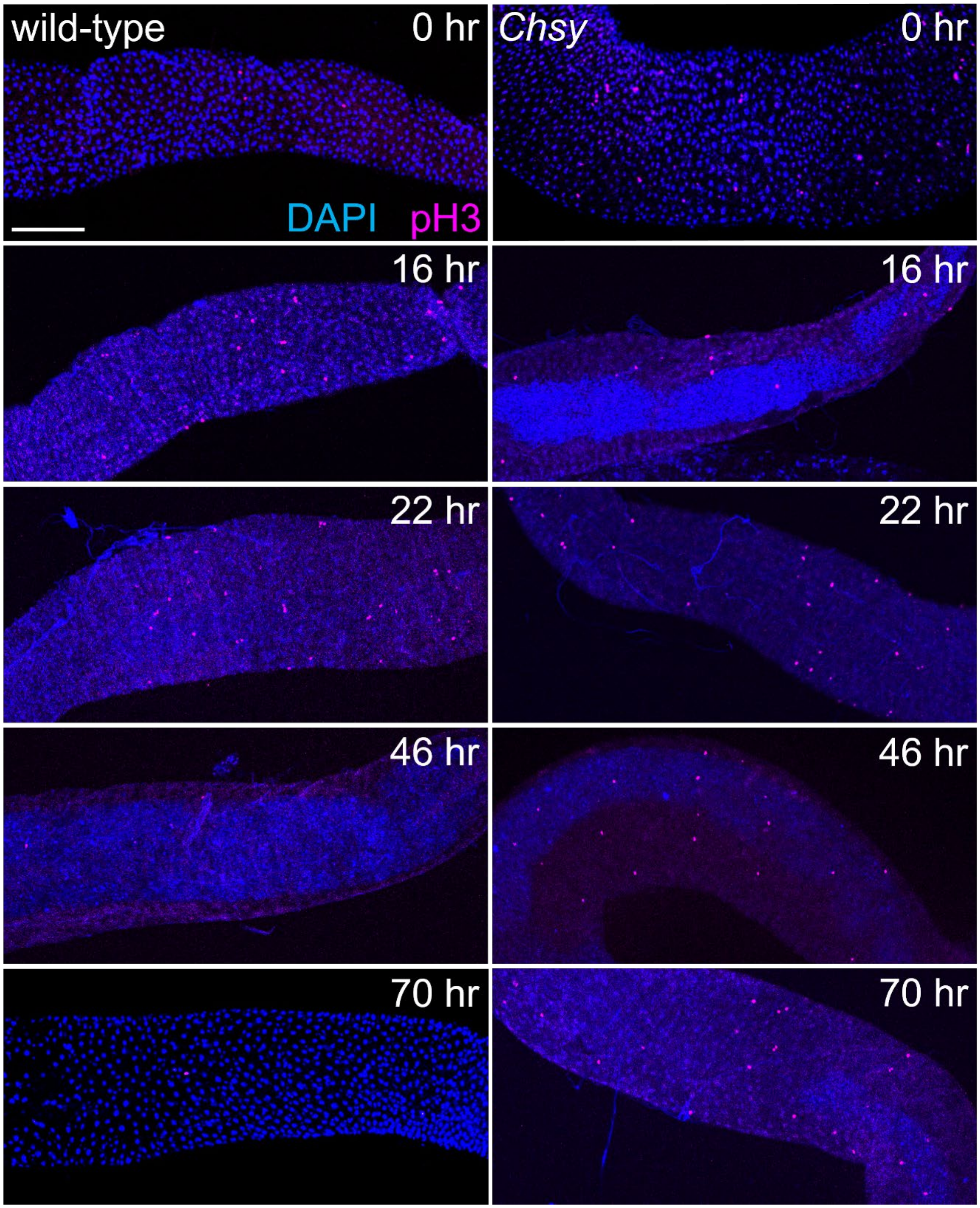

S4 Fig. ISC proliferation in *Chsy* mutants during regeneration.
Wild-type (left column) and *Chsy* (right column) midguts were stained with DAPI (blue) and anti-pH3 (magenta) at indicated time points during regeneration. The number of pH3-positive cells decreased within 70 hours after the beginning of infection in wild-type, whereas this reduction was not observed in *Chsy* mutants. Quantification of these data is shown in Fig 4G. Scale bar: 100 µm.
