# Supplementary material for: Chondroitin sulfate regulates proliferation of Drosophila intestinal stem cells
Source: PLoS Genet. 2025 May 9;21(5):e1011686. doi: 10.1371/journal.pgen.1011686 (PMC12063844; doi:10.1371/journal.pgen.1011686)
Supplement: S5 Fig — (PDF) [file pgen.1011686.s007.pdf]

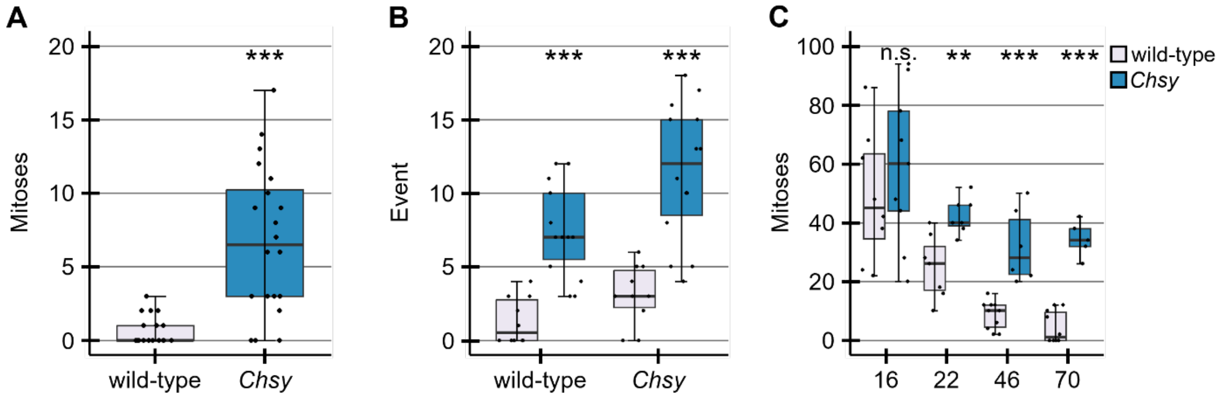

S5 Fig. *Chsy* mutant males show the consistent phenotypes with females.
(A) Quantification of pH3-positive cells in the midguts of wild-type and *Chsy* mutant males under homeostatic conditions. The number of pH3-positive cells was significantly increased in *Chsy* mutant males. (B) Quantification of longitudinal muscle defects in *Chsy* mutant males. Muscle discontinuity and sprouting were increased in *Chsy* male. (C) Regeneration assay for *Chsy* males. *Chsy* mutant ISCs failed to properly downregulate mitotic activity at the end of regeneration. Across these experiments, *Chsy* mutant males exhibited the consistent phenotypes with females. Boxes indicate the 25-75th percentiles, and the median is marked with a line. The whiskers extend to the highest and lowest values within 1.5 times the interquartile range. $^{**}P<0.01$; $^{***}P<0.001$; n.s., not significant (two-sided, unpaired t-test).
