# Supplementary material for: Chondroitin sulfate regulates proliferation of Drosophila intestinal stem cells
Source: PLoS Genet. 2025 May 9;21(5):e1011686. doi: 10.1371/journal.pgen.1011686 (PMC12063844; doi:10.1371/journal.pgen.1011686)
Supplement: S6 Fig — (PDF) [file pgen.1011686.s008.pdf]

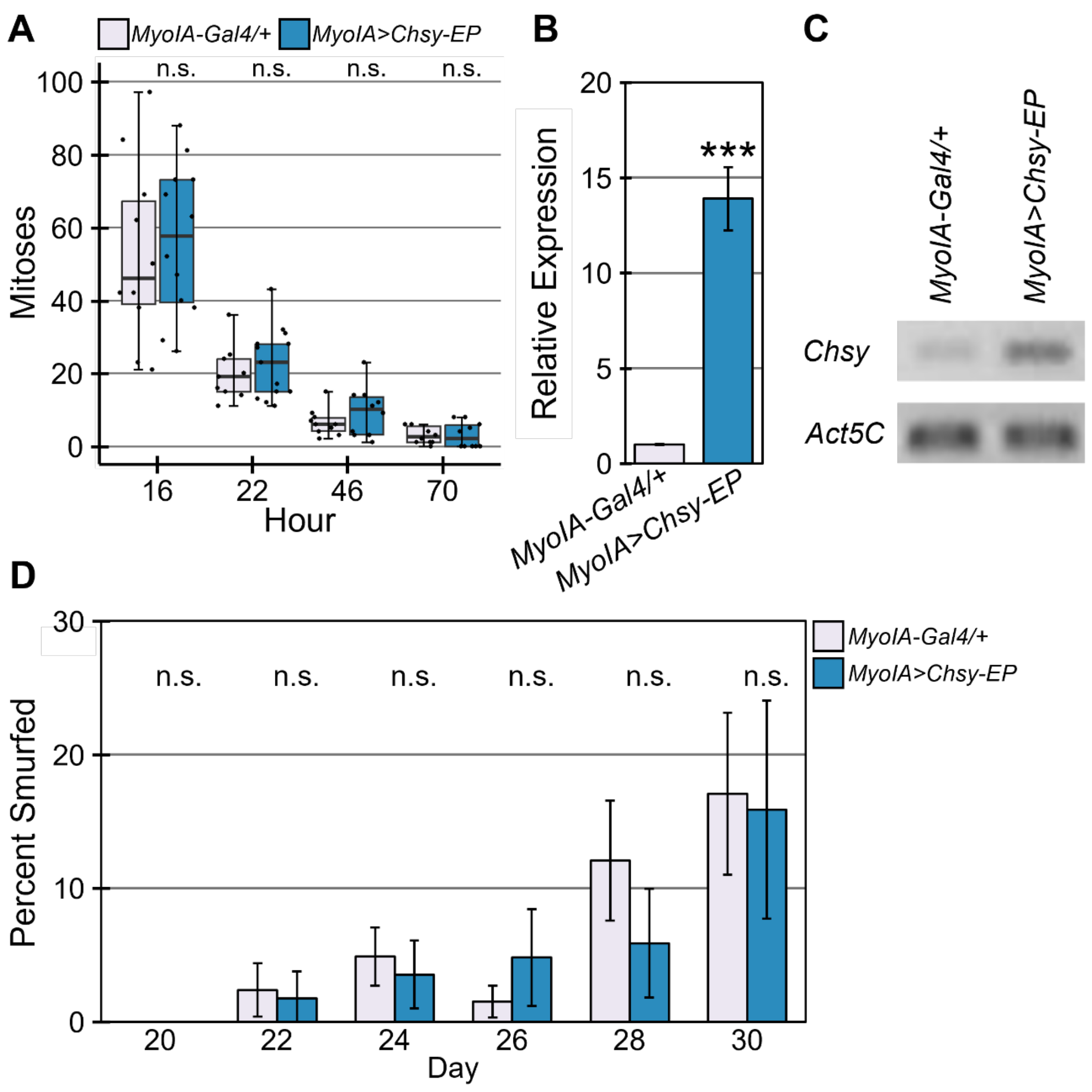

S6 Fig. The effects of *Chsy* overexpression in the midgut.

(A) Quantification of pH3-positive cells in midguts from *Chsy*-overexpressing (*MyoIA>Chsy-EP*) and control (*MyoIA-Gal4/+*) flies during regeneration. Midguts were dissected at 16, 22, 46, and 70 hours after the beginning of *Ecc15* infection. (B and C) RT-qPCR quantification of *Chsy* mRNA in midguts from *MyoIA>Chsy-EP* and *MyoIA-Gal4/+* flies. *Chsy* mRNA levels were significantly increased in *MyoIA>Chsy-EP*. The average expression levels of *Chsy* were normalized to 1.0. (D) Quantification of barrier integrity assay of *MyoIA>Chsy-EP*. None of the flies smurfed by 20 days after eclosion (13 days after starting the assay). Flies with *Chsy* overexpression in ECs (*MyoIA>Chsy-EP*) did not show any significant difference percentages of smurfed population during aging compared to control animals (*MyoIA-Gal4/+*). Boxes indicate the 25-75th percentiles, and the median is marked with a line. The whiskers extend to the highest and lowest values within 1.5 times the interquartile range. ***P<0.001; n.s., not significant (two-sided, unpaired t-test).
