# Supplementary material for: Chondroitin sulfate regulates proliferation of Drosophila intestinal stem cells
Source: PLoS Genet. 2025 May 9;21(5):e1011686. doi: 10.1371/journal.pgen.1011686 (PMC12063844; doi:10.1371/journal.pgen.1011686)
Supplement: S7 Fig — (PDF) [file pgen.1011686.s009.pdf]

A

GCGGCAATTTAGCTACGCCTTGG

B

1 MRRPFTALNRNHYFVIGLLLGLLLSWHIPEDIWEEECPEEAAENLLIERF 50
51 GQEFEPHLNLINKPLAAKKPVKNVIRPRYYSSELGIREKLFIGVMTSQEH 100
101 INTFATAFNRTTAHLVNKIKFFIYADSVKTNYKLKNIVGFTDTRESRRPF 150
151 HVVKYIADNYLDEYDYFLLVPDTVYVDARKLVKLLYHMSITFDLYMGGAR 200
201 IGLDPSGGGASADGQSNEPPANEEEAPGASDRNYCSLEAGILLSSSVIRK 250
251 MRNNLERCVRIGSTSDHSVNIGRCVKYASRVAGCQESFQGMRQFSYALDA 300
301 PGRRHREFSELAKEEAFRNASTVYPVQTPEDFYRLHAYYSKHHLEKVQER 350
351 GYALEQKSYRIANGSISNKILEIRWPLGVPPPSAPETRHDILTWQLLNGT 400
401 HNFLPNGNAEHAVATLSRIEAQDFAKVLEIALQYAALKHPRLSYHSLHSA 450
451 YRKFDATRGMDYQLHLNLQEGSGRSRRLVIKSFEVVKPLGRVEVVPSPYV 500
501 TESTRIAMLVPAFEHQVPDALLFVEQYERICMQNQDNTFLLLIFMYRLES 550
551 PSKGDEDPFKALKTLALDLSSKYKTDGSRIAWVSIRLPEQLSEPVDPQSW 600
601 LLHASMYGPRQLLSLVVADLALPKLGLESLVLLATPGMVFKADFLNRVRM 650
651 NTIQGFQVYAPIGFQMYPCRWAHFCRECDTCDVSQSSGYFDRHNHDVIAF 700
701 YSRDYVQARKLLHPQGLPIIRSDLDIDQLLLQPGEETRPPGVESILDMFV 750
751 AAQHSVHILRGVEPNLRFGQDVRNHLARGGTLPQSVPERCGREQCIHLAS 800
801 RKQIGDAIIRYEDKSILHK 819

S7 Fig. Generation of *Chpf*^{424} allele.

(A) The gRNA sequence. The CRISPR/Cas9-mediated mutagenesis induced a deletion of 11 base pairs (AGCTACGCCTT, underlined). This allele was referred to as *Chpf*^{424}. (B) The entire amino acid sequence of wild-type Chpf (819 amino acids) is shown. In *Chpf*^{424}, the 11 bp-deletion caused a frame shift, resulting in a truncated protein (amino acid number 1-294, underlined).
