# Supplementary material for: Chondroitin sulfate regulates proliferation of Drosophila intestinal stem cells
Source: PLoS Genet. 2025 May 9;21(5):e1011686. doi: 10.1371/journal.pgen.1011686 (PMC12063844; doi:10.1371/journal.pgen.1011686)
Supplement: S8 Fig — (PDF) [file pgen.1011686.s010.pdf]

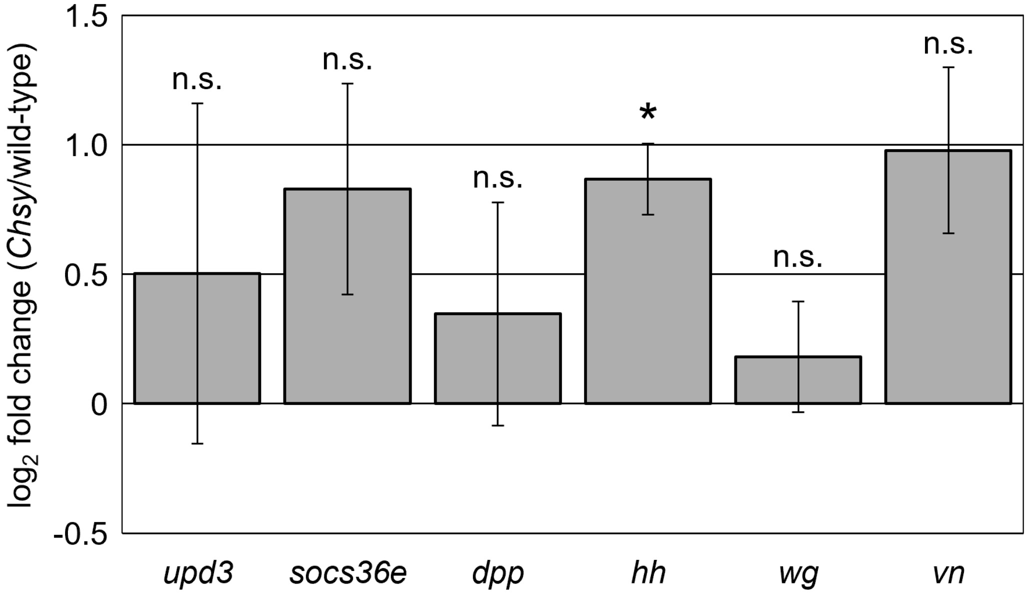

S8 Fig. Mitogenic signaling is upregulated in the *Chsy* mutants.
RT-qPCR analysis of the JAK/STAT (*upd3*, *socs36e*), BMP (*dpp*), Hedgehog (*hh*), Wingless (*wg*), and EGFR (*vn*) pathways in *Chsy* midguts under homeostatic conditions. RNA samples of wild-type and *Chsy* whole midguts were acquired from flies 5 days after eclosion. Error bars represent mean±s.e.m. (n=4). **P*<0.05; n.s., not significant (two-sided, unpaired t-test).
